# Comparison Between Concentrated Autologous Bone Marrow Aspirate Transplantation as a Hip Preserving Surgery and Natural Course in Idiopathic Osteonecrosis of the Femoral Head

**DOI:** 10.7759/cureus.24658

**Published:** 2022-05-02

**Authors:** Yohei Tomaru, Tomokazu Yoshioka, Hisashi Sugaya, Hiroshi Kumagai, Katsuya Aoto, Hiroshi Wada, Hiroshi Akaogi, Masashi Yamazaki, Hajime Mishima

**Affiliations:** 1 Department of Orthopedic Surgery, University of Tsukuba, Tsukuba, JPN; 2 Department of Orthopedic Surgery, Chiba Child and Adult Orthopedic Clinic, Chiba, JPN; 3 Department of Orthopedic Surgery, Tsukuba University of Technology, Tsukuba, JPN

**Keywords:** core decompression, mesenchymal stem cells, concentrated autologous bone marrow aspirate transplantation, joint-preserving surgery, osteonecrosis of the femoral head

## Abstract

Purpose

The purpose is to compare the therapeutic efficacy of concentrated autologous bone marrow aspirate transplantation (CABMAT) with that of observation alone for osteonecrosis of the femoral head (ONFH).

Methods

This single-center study included patients with idiopathic ONFH that were either treated with CABMAT (CABMAT group) or managed through observation alone (observation group) over a >2-year follow-up period. The Japanese Investigation Committee classification was used to diagnose and classify ONFH. The collapse rates for stages 1 and 2 ONFH (i.e., pre-collapse stages) and the THA conversion rates were compared between the CABMAT and observation groups.

Results

The CABMAT and observation groups comprised 232 (mean follow-up: 8.2 years) and 106 (mean follow-up: 6.0 years) patients, respectively. No significant intergroup differences were noted in the stages, types, and associated factors of ONFH. The collapse rates for pre-collapse stages in the CABMAT and observation groups were 67.1% and 65.3%, respectively. For stage 1, the collapse rates were significantly lower in the observation group than in the CABMAT group (p<0.05). The overall THA conversion rates in the CABMAT and observation groups were 24.3% and 41.5%, respectively (p<0.0001). For ONFH of stages 3A and 3B (collapse stages), the THA conversion rates were significantly lower in the CABMAT group (p<0.05).

Conclusion

Collapse rates were significantly higher for stage 1 ONFH; for collapse stages, the THA conversion rates were significantly lower in the CABMAT group than in the observation group. Therefore, observation and CABMAT are recommended for ONFH of stage 1 and for ONFH of higher stages, respectively.

## Introduction

Osteonecrosis of the femoral head (ONFH) occurs in highly active young individuals and has shown an increased incidence in recent years [1]. The outcomes of total hip arthroplasty (THA) are improving because of developments in surgical techniques and implant materials [2]. However, unlike osteoarthritis in elderly patients, ONFH in young patients is not a good indication for THA for various reasons, such as high physical activity, risk of dislocation, and long survival expectancy. Therefore, joint preservation is preferred for ONFH in younger patients [3].

During the natural course of ONFH, extensive necrosis often results in femoral head collapse [4]. It is important to prevent this collapse (and the consequent transition to THA) in cases that appear to have a poor prognosis. Various joint-preserving procedures have been reported, with core decompression (CD) as one of the most common treatments. Furthermore, a meta-analysis revealed that using a combination of bone marrow mesenchymal stem cells (MSCs) and CD improved therapeutic efficacy [5].

Between 2002 and 2015, patients with ONFH at our institution were treated with concentrated autologous bone marrow aspirate transplantation (CABMAT) [6]. Because CABMAT is difficult to perform in Japan because of restrictions imposed by regenerative medicine law, ONFH was managed through simple observation alone both before (2001) and after this period. Although many studies have demonstrated the therapeutic efficacy of bone marrow aspirate concentrate, the patients’ background characteristics and methods of bone marrow aspiration, concentration, and transplantation vary. Despite these factors, CABMAT is simple, single-step, and inexpensive, and thus its therapeutic efficacy should be further evaluated [6]. However, the efficacy of CABMAT with that of observation alone has not been compared. We hypothesized that CABMAT prevents femoral head collapse secondary to ONFH, thereby preventing the consequent conversion to THA. This study was conducted to compare the therapeutic efficacies of CABMAT and observation in patients with ONFH.

## Materials and methods

The study design was approved by the Institutional Ethical Review Committee. Written informed consent was obtained from all included patients. The inclusion criteria were as follows: 1) patients with non-traumatic idiopathic ONFH treated using CABMAT (CABMAT group) or managed through observation alone (observation group) at our institution between December 2001 and December 2018 and 2) patients followed up for a period of >2 years.

From 2002 to 2015, ONFH was mainly treated by CABMAT; ONFH stages 1, 2, 3A, 3B, and 4 were indications for CABMAT. During other years, i.e., in 2001 and from 2015 onward, patients with all stages and types of ONFH were subjected to observation alone. The treatment method was decided by three or more orthopedic surgeons as well as based on the patient’s preference and whether the patient had been examined during the period when CABMAT was performed at our institution (2002-2015). Patients who did not desire CABMAT due to the duration of postoperative non-weight bearing and the possibility of prolonged pain compared to THA were excluded from the CABMAT group.

The Japanese Investigation Committee classification, which is based on the stage and type, was used to diagnose and classify ONFH [7]. The stage classification is based on the findings of a plain radiograph: stage 1, no abnormality; stage 2, osteosclerosis without femoral head collapse; stage 3A, the femoral head collapse of less than 3 mm; stage 3B, the femoral head collapse of more than 3 mm; and stage 4, osteoarthritis. The type classification is based on the extent of necrosis, as determined using magnetic resonance imaging: in type A, necrosis does not exceed one-third of the loading surface of the joint; in type B, necrosis does not exceed two-thirds of the loading surface of the joint; type C1, necrosis does not exceed the acetabular margin; and type C2, necrosis exceeds the acetabular margin.

CABMAT was performed according to an established procedure described previously [6]. Under the general anesthesia, the patient was positioned on the traction table (Yuno, Getinge, Sweden). Approximately 300 mL of bone marrow was aspirated from the iliac crest. The sample was centrifuged twice using a general blood bag (Terumo, Tokyo, Japan) in a general centrifuge (Kubota, Japan 9800, Kubota) to obtain the buffy coat; approximately 30 mL of the buffy coat was extracted. CD was performed using a 4.8-mm diameter trephine (Iso Medical Systems, Tokyo, Japan). This instrument was inserted into the center of the necrotic site and transplantation was performed. The positioning of the instrument was monitored with biplane fluoroscopy.

In addition to MSCs, the buffy coat contained basic fibroblast growth factor, platelet-derived growth factor-BB, vascular endothelial growth factor, transforming growth factor-β1, and bone morphogenetic protein-2 [8]. Weight-bearing was limited for 6 weeks after the surgery, while non-weight bearing was not limited. Orthotic therapy was not performed.

Anteroposterior plain radiographs were obtained during each hospital visit. The extent of the collapse was evaluated using an overlay circle as described by Aaron et al. [9]. An increase in the collapse by ≥1 mm was defined as collapse progression. The date of the onset of the collapse was defined as the date on which the collapse was first identified on plain radiography. By setting collapse occurrence as the endpoint, the survival rates in the pre-collapse stages (stages 1 and 2) in the CABMAT and observation groups were compared using the log-rank test. The collapse progression distance was calculated by subtracting the collapse distance of the femoral head at the initial examination from the collapse distance of the femoral head at the most recent follow-up. These values were compared between the CABMAT and observation groups using a t-test.

Indication of THA in this study was patients’ preference, pain, and radiological factors (collapse, osteoarthritis). Using THA conversion as the endpoint, the survival rate was compared between the CABMAT and observation groups using the log-rank test.

Multivariable analyses were performed to evaluate the factors predictive of collapse and THA conversion. Femoral head collapse and THA were set as the objective variables, whereas age, sex, associated factors, follow-up period, ONFH type and stage at initial visit, and treatment (CABMAT or observation) were considered as explanatory variables.

All statistical analyses were performed using the statistical program R (http://cran.r-project.org, The R Project for Statistical Computing, Vienna, Austria). Statistical significance was set at p < 0.05.

## Results

Among the 405 patients (677 hips), 338 patients (558 hips) were followed up for >2 years and were included in this study (follow-up rate: 83%). Among these, 232 (387 hips) and 106 (171 hips) patients were categorized into the CABMAT and observed groups, respectively. The mean ages at the initial visit were 40.1 and 48.9 years in the CABMAT and observation groups, respectively. The mean follow-up periods were 8.2 and 6.0 years in the CABMAT and observation groups, respectively.

Data on the patient characteristics, follow-up periods, and associated factors for both groups are shown in Table 1.

**Table 1:** Characteristics of patients in the two groups *p<0.01 (t-test), **: p<0.001 (Wilcoxon exact test), CABMAT: concentrated autologous bone marrow aspirate transplantation

Characteristics	CABMAT group	Observation group
Patient number	232	106
Joint number	387	171
Age	40.1 (range 14.3–77.2)	48.9* (range 14.3–84.4)
Male	213 (55%)	74 (43%)
Female	174 (45%)	97 (57%)
Follow-up period (year)	8.2** (range 2.1–17.6)	6.0 (range 2.0–19.2)
Associated factors		
Steroid	280 (72%)	137 (80%)
Alcohol	76 (20%)	22 (13%)
Idiopathic	31 (8.0%)	12 (7.0%)

The mean age was significantly higher in the observation group than in the CABMAT group (p < 0.001, t-test). The mean follow-up period was significantly longer in the CABMAT group than in the observation group (p < 0.001, Wilcoxon exact test). There were no significant differences in the preoperative types and stages of ONFH and ONFH-associated factors between the two groups. The group-wise distributions of the types and stages of ONFH at the initial visit and of the collapse and THA conversion rates for each type and stage are presented in Tables 2, 3.

**Table 2:** Collapse rate, THA conversion rate of each types and stages in CABMAT group a. Type and stage at the initial visit in the CABMAT group. b. Collapse rate (%) in each type and pre-collapsed stages. c. THA conversion rate (%) in each type and stage. THA: total hip arthroplasty, CABMAT: concentrated autologous bone marrow aspirate transplantation

a			Types				
		Stages	A	B	C1	C2	Total
	Stages	1	3	18	57	28	106
		2	1	11	61	49	122
		3A	-	3	36	62	101
		3B	-	-	10	35	45
		4	-	-	4	9	13
		Total	4	32	168	183	387
b			Types				
		Stages	A	B	C1	C2	Total
	Stages	1	33.3	44.4	54.4	75.0	57.5
		2	0.0	18.2	52.5	79.6	59.8
							34.6
c			Types				
		Stages	A	B	C1	C2	Total
	Stages	1	0.0	5.6	26.3	42.9	26.4
		2	0.0	0.0	16.4	26.5	18.9
		3A	-	-	75.0	55.6	61.5
		3B	-	33.3	2.8	27.4	18.8
		4	-	-	0.0	45.7	35.6
		Total	0.0	6.3	17.3	34.4	24.3

**Table 3:** Collapse rate, THA conversion rate of each types and stages in observation group a. Types and stages at the initial visit in the observation group. b. Collapse rate (%) in each type and pre-collapsed stages. c. THA conversion rate (%) in each preoperative types and stages. THA: total hip arthroplasty

a			Types				
		Stages	A	B	C1	C2	Total
	stages	1	1	18	18	10	47
		2	-	6	22	23	51
		3A	-	-	11	31	42
		3B	-	-	3	19	22
		4	-	-	3	6	9
		Total	1	24	57	89	171
b			Types				
		Stages	A	B	C1	C2	Total
	stages	1	100.0	22.2	22.2	70.0	34.0
		2	-	33.3	45.5	69.6	54.9
							44.9
c			Types				
		Stages	A	B	C1	C2	Total
	stages	1	100.0	16.7	16.7	50.0	25.5
		2	-	16.7	13.6	34.8	23.5
		3A	-	-	45.5	74.2	66.7
		3B	-	-	100.0	63.2	68.2
		4	-	-	66.7	33.3	44.4
		Total	100.0	16.7	28.1	56.2	41.5

**Figure 1:**
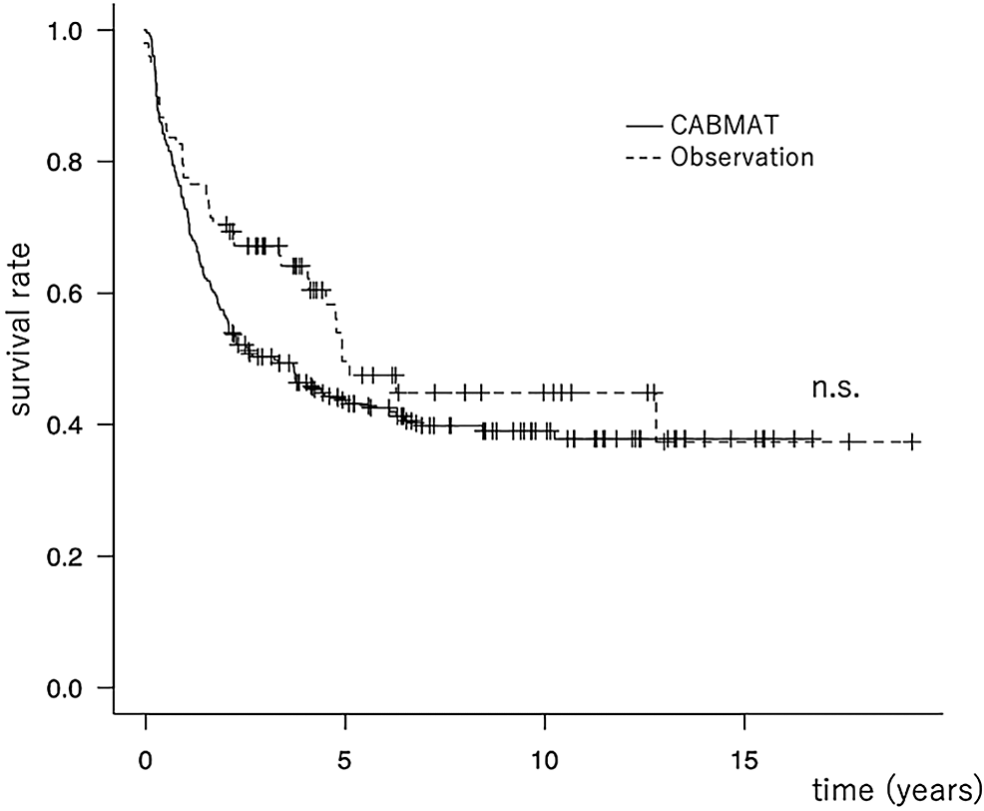
Survival curve in the pre-collapse stages (endpoint, collapse; n.s., not significant) CABMAT: concentrated autologous bone marrow aspirate transplantation

The mean durations until collapse were 1.6 and 2.0 years in the CABMAT and observation groups, respectively. Using collapse as the endpoint for the pre-collapse stages revealed no significant differences in the survival rates between the two groups (p > 0.05, log-rank test; Figure 1).

**Figure 2:**
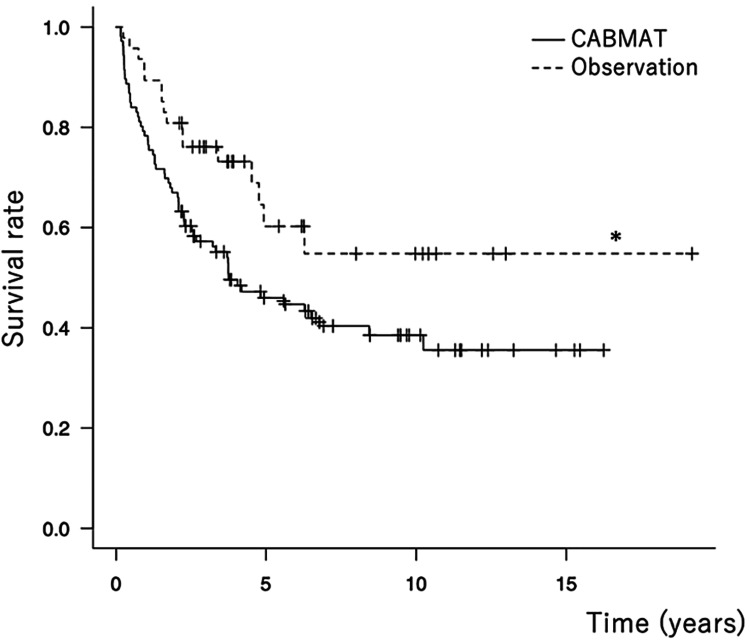
Survival curve for stage 1 (endpoint, collapse; *, p<0.05) CABMAT: concentrated autologous bone marrow aspirate transplantation

However, for stage 1, the survival rate was significantly higher in the observation group than in the CABMAT group (p < 0.05, log-rank test; Figure 2).

**Figure 3:**
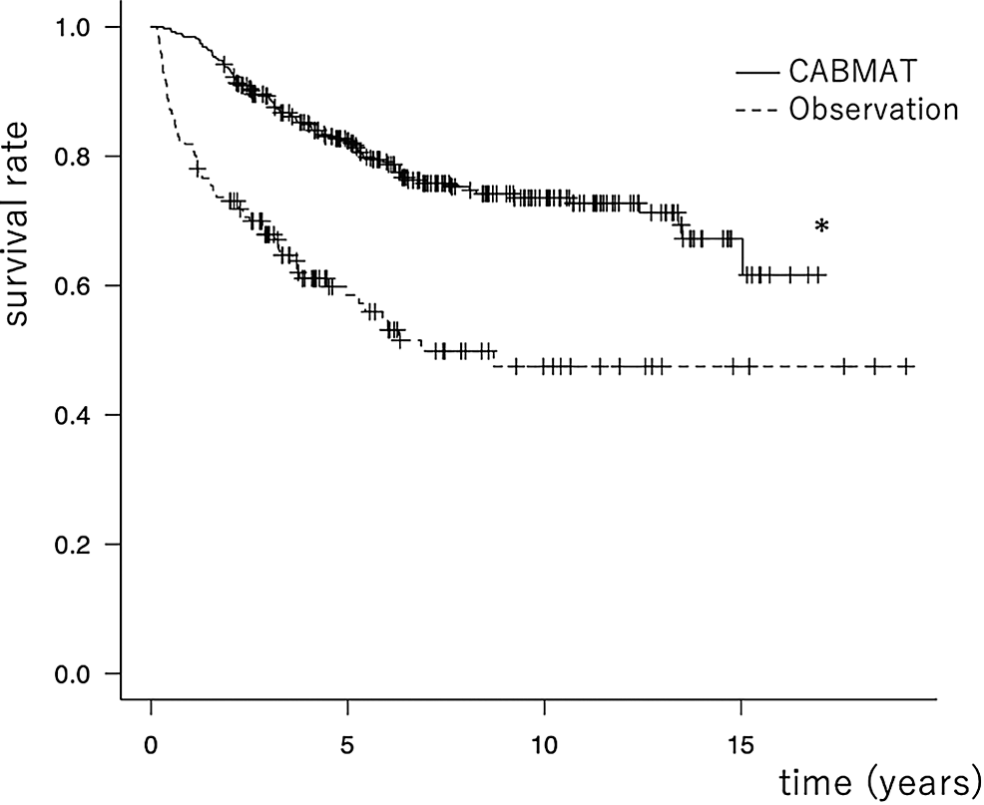
Survival curve in the overall stages (endpoint, conversion to total hip arthroplasty; *, p<0.001) CABMAT: concentrated autologous bone marrow aspirate transplantation

The type-wise collapse rates did not differ significantly between the two groups (p > 0.05, log rank test). The overall mean collapse progression distances were 3.0 and 2.5 mm in the CABMAT and observation groups, respectively (p > 0.05, t-test). For the pre-collapse stages, the mean collapse progression distances were 3.0 and 1.9 mm in the CABMAT and observation groups, respectively (p < 0.05, t-test). For the collapse stages (i.e., stages 3A, 3B, and 4), the mean collapse progression distances were 3.0 and 3.2 mm in the CABMAT and observation groups, respectively (p > 0.05, t-test). For the pre-collapse stages, the collapse progression was significantly higher in the CABMAT group than in the observation group. For the collapse stages, the collapse progression was lower in the CABMAT group than in the observation group but this difference was not significant. The THA conversion rates were 24.3% (94/387 hips; mean time to conversion: 3.9 years) and 41.5% (71/171 hips; mean time to conversion: 1.9 years) in the CABMAT and observation groups, respectively. Using THA conversion as the endpoint, the overall survival rate was significantly higher in the CABMAT group than in the observation group (p < 0.0001, log-rank test; Figure 3).

For stages 3A and 3B and for types C1 and C2, the survival rates were significantly higher in the CABMAT group than in the observation group; however, no significant intergroup differences were observed for stages 1, 2, and 4 and for types A and B (log rank test).

Multivariate analysis revealed that the occurrence of collapse was significantly correlated with associated factors (p < 0.05) and the type of ONFH (p < 0.001) at the initial visit. The age (p < 0.001), type of ONFH (p < 0.001), ONFH stage at initial visit (p < 0.05), and treatment (CABMAT or observation; p < 0.01) were also significantly associated with THA conversion.

Regarding complications, after CABMAT, no deep infections and oncogenesis occurred, but in one case, subtrochanteric femur fracture happened, which required open reduction and internal fixation.

## Discussion

There were no significant differences in the overall collapse rates between the CABMAT and observation groups in the pre-collapse stages. According to subgroup analyses based on the stages and types of ONFH, the collapse rate was significantly higher at stage 1 in the CABMAT group than in stage 1 in the observation group. The overall THA conversion rate was significantly lower in the CABMAT group than in the observation group. Furthermore, the THA conversion rates for stages 3A and 3B and types C1 and C2 were significantly lower in the CABMAT group than in the observation group; however, there were no significant intergroup differences for other stages and types. Based on these findings, we recommend careful observation in relatively mild cases (i.e., cases with stages 1 and 2 and types A and B) and CABMAT in severe cases (stages 3A and 3B and types C1 and C2).

The causes of idiopathic ONFH are considered multifactorial. Corticosteroid usage and alcohol abuse are known risk factors [1]. Osteonecrosis can also be caused by the obstruction of the blood flow due to thrombosis, fat embolization, intraosseous hypertension, or endothelial cell dysfunction, followed by MSC dysfunction and bone cell necrosis [10,11]. In addition, studies have reported relationships between ONFH and growth factors, such as the vascular endothelial growth factor, basic fibroblast growth factor, transforming growth factor β1, and bone morphogenic protein-2 [12,13]. The buffy coat obtained from the bone marrow aspirate contained not only MSCs and hematopoietic cells, but also the growth factors listed above [8]. Using a rabbit model, Sugaya et al. found that implanted MSCs could differentiate into osteoblasts at the transplanted site [14]. Thus, in our study, the transplanted MSCs likely differentiated into osteoblasts, and the accompanying growth factors contributed to bone regeneration after necrosis.

Several joint-preserving treatments have been developed. CD is among the most common joint-preserving surgeries. CD and multiple drilling result in a reduction of the intraosseous pressure, and the paracrine effect of healthy bone is a tenet for CD and multiple drilling. A wide range of success rates has been reported (29-90%). Favorable outcomes have been reported for small- to medium-sized necrotic lesions in the pre-collapse stages but not in the collapse stages [15].

At stage 1, the collapse rate was significantly higher in the CABMAT group than in the observation group. Although the precise reason for this is unclear, CD may have decreased the mechanical strength and increased the collapse rate. Recently, several studies reported favorable outcomes for multiple drilling rather than CD [16]. Considering the mechanical strength of the femoral head, multiple drilling may be more advantageous than a CD.

For stages 3A, 3B, and 4, the THA conversion rate was significantly lower in the CABMAT group; collapse progression was also lower in the CABMAT group but not significantly. CABMAT may have reduced further collapse progression and subsequent THA conversion in the collapse stages. In addition to the extent of collapse, pain is an important factor. CD may have contributed to pain relief and prevented THA conversion.

Recently, several regenerative medicines have been used in combination with CD. Several reports, including meta-analyses, demonstrated the increased efficacies of implantation of bone marrow-derived MSCs when combined with CD [5,17]. In contrast, one prospective randomized study reported no benefit from bone marrow transplantation [18]. Gangji et al. observed collapse rates of 15.8% (3/19) and 72.7% (8/11) in bone marrow transplantation and CD alone groups, respectively [19], supporting the efficacy of bone marrow transplantation. Hernigou et al. reported that in 534 hips treated with bone marrow transplantation, the collapse and THA conversion rates during 13 years of follow-up were 30% (160/534) and 18% (96/534), respectively [20]. Mao et al. reported that for bone marrow transplantation via the medial circumflex femoral artery, the THA conversion and radiological progression rates were 7.7% (6/78) and 43.6% (34/78), respectively [21]. Several studies found favorable outcomes for vascularized bone grafts. According to Zhao et al., the survival rate after vascularized iliac bone grafting was 88% in 56 hips during five years of follow-up [22]. Feng et al. reported a 100% survival rate in 30 hips following fibula vascularized bone grafting during 2.2 years of follow-up [23]. In the present study, over a mean follow-up period of 3.9 years, the collapse rate in the pre-collapse stages and THA conversion rate in the CABMAT group were 67.1% (153/228 hips) and 24.3% (94/387 hips), respectively. Favorable outcomes using joint-preserving techniques have been reported, including some studies showing better outcomes than in our study. ONFH is complex in terms of its severity and etiology, making it difficult to compare treatment outcomes. We performed bone marrow transplantation not only for stages 1 and 2 but also for stages 3 and 4, which may have influenced the results.

In addition, various methods are used to extract and concentrate MSCs from the bone marrow. The quantities of MSCs and growth factors included in the bone marrow to be transplanted are also variable, which may have affected our results. We used a general blood bag and centrifuge for concentration measurements as a simple and low-cost method. Compared with osteotomy and visualized bone grafting, CABMAT is relatively less invasive, less costly, and easier to perform.

This study had some limitations. First, to verify the efficacy of CABMAT, the CD group should have been used as the control group. However, in our institution, the CD was not performed as the only procedure; therefore, the observation group was used as the control group. Second, because this was a retrospective study, accurate data on clinical symptoms were unavailable. Third, Patients with a strong desire for joint preservation tended to receive CABMAT, which may have contributed to the lower rate of THA conversion compared to the observation group.

## Conclusions

In stage 1 ONFH, the collapse rate was significantly higher and the THA conversion rate was significantly lower in the CABMAT group than in the observation group. Based on this finding, careful follow-up is recommended for Stage 1 ONFH, whereas CABMAT is recommended for the other stages of collapse. CABMAT may have prevented a further major femoral head collapse in these stages, which otherwise would have led to THA conversion.
